# Disability and its association with sociodemographic factors among elderly persons residing in an urban resettlement colony, New Delhi, India

**DOI:** 10.1371/journal.pone.0222992

**Published:** 2019-09-24

**Authors:** Anil Kumar Goswami, Ramadass S., Mani Kalaivani, Baridalyne Nongkynrih, Shashi Kant, Sanjeev Kumar Gupta

**Affiliations:** 1 Centre for Community Medicine, All India Institute of Medical Sciences, New Delhi, India; 2 Department of Biostatistics, All India Institute of Medical Sciences, New Delhi, India; Institute of Economic Growth, INDIA

## Abstract

Disability prevents an individual from performing to the fullest potential. It is multidimensional. Disability may be physical, mental, social, personal, and environmental or a combination of these. The elderly experience an increased burden of disability, especially in areas where there are limited resources and rapid urbanization. Comparison of reported disability is difficult because several definitions and scales are in use. We used the World Health Organization Disability Assessment Schedule version 2.0 (WHODAS 2.0) to study the prevalence of disability, and its association with sociodemographic factors among elderly persons residing in an urban resettlement colony, New Delhi, India. The WHODAS 2.0 provides continuous summary scores, where higher scores indicate higher disability, and vice versa. Elderly persons aged 60 years and above were selected by simple random sampling in this community-based cross-sectional study. Trained interviewers administered the semi-structured interview schedule and WHODAS 2.0. The prevalence of disability was 7.4% (5.8% - 9.3%) among the 931 participants. The prevalence was higher among females than males. Female sex, elderly aged 70 years and above, and those who were illiterate had increased risk of higher disability scores. Participants who were in government or private service had 50% decreased risk of having higher disability scores. The burden of disability was high among elderly persons residing in this resettlement colony. Community-based holistic interventions are required to mitigate the disability, and to improve the functioning of elderly persons.

## Introduction

Disability is multidimensional, and complex to measure. An individual may be disabled temporarily or permanently at any point in time. Those who survive to old age experience an increased burden of disability. The International Classification of Functioning, Disability and Health (ICF) promotes disability as a “bio-psycho-social model” [1]. It defines disability as an umbrella term for impairments, activity limitations and participation restrictions, referring to the negative aspects of the interaction between an individual (with a health condition) and that individual’s contextual factors (environmental and personal factors) [2]. Persons aged 60 years and above were defined as elderly by the National Policy on Older Persons, Government of India [3]. As per the national census 2011, elderly persons accounted for 8.6% of the overall population. The proportion of elderly persons in India is projected to increase to 12.17% of the total population in the year 2026 [4]. Disability was measured by the National Sample Survey Organisation, and also during Census 2001 and 2011 [5–7]. The scales used were essentially based on the medical model of disability and were specific to certain medical conditions. In census 2011, 5.1% of the elderly were either physically or mentally disabled [8]. The community-based cross-sectional studies among elderly persons in India had used scales that measured disability as impairment or activity limitation or participation restriction [9]. These scales were Rapid Disability Rating scale-2, Barthels Activity of Daily Living score, Instrumental Activity of Daily Living Score, Standard Health Assessment Questionnaire. The Washington Group on Disability developed a short and extended set of questions for measuring disability and functioning [10]. The proportion of disability measured by this scale among the elderly persons ranged from 16.2% to 87.5% [11–18]. The ICF was developed to standardise the measurement of disability, and to promote the multidimensional model of disability [19]. It is impractical to measure the disability using the ICF, because it is a classification system that provides a standard for health and disability statistics [20]. The usage of complicated terminology and subjectivity of the assessors to code were the major shortcomings of the ICF [21]. To overcome this, the World Health Organisation developed the World Health Organisation Disability Assessment Schedule 2.0 (WHODAS 2.0) to reflect the concept of the ICF [22]. India is a signatory to the development process of the WHODAS 2.0.

India is experiencing rapid urbanisation. The population living in urban areas increased from 27.81% in the 2001 Census to 31.16% in the 2011 Census [23]. Unplanned urban growth is associated with increase in urban slum population and environmental degradation [24]. Population demands were beyond the environmental service capacity, in areas of drinking water, sanitation, and waste disposal and treatment. Access to housing, sanitation, health care services, food expenditure are the important factors for explaining urban poverty in India [25, 26]. Elderly persons experience more difficulty because of inaccessible cities, age-unfriendly environment, and increase in non-communicable diseases [27].

There is little evidence of the burden of disability among elderly persons in urban areas using the multidimensional concept of disability. We aimed to study the prevalence of disability using the WHODAS 2.0, and its association with socio-demographic factors in an urban resettlement colony of Delhi.

## Methods

This community-based cross-sectional study was conducted between February and May 2018 in an urban resettlement colony, located in New Delhi, India. The resettlement colony had an approximate total population of 36,500 including approximately 2900 elderly persons. All elderly persons aged 60 years and above, and residing in the study area for at least last six months were eligible for inclusion in the study. Participants who could not communicate and comprehend were excluded. A sample of 1006 elderly persons was required to estimate the prevalence of disability of 4.5% with 30% relative precision and 10% non-response [8]. These elderly persons were selected by simple random sampling. Two specially recruited non-specialist graduate interviewers made house-to-house visits. Interviewers were extensively trained in the administration of semi-structured interview schedule and World Health Organisation Disability Assessment Schedule 2.0 (WHODAS 2.0) 36-item interviewer version in the local vernacular language [22]. The Principal Investigator supervised the interviews in the field. Random checks were made every week thereafter. A maximum of three visits were made to the selected elderly persons home to establish the eligibility. All those who gave written informed consent to participate were administered the semi-structured interview schedule and WHODAS 2.0. In the semi-structured interview schedule, we recorded the demographic and the socioeconomic variables. Age of the participant was formally established during interview from stated age or official documentation. A participant was considered economically independent if his/her source of personal income or any monetary benefit from the social welfare scheme was perceived to be sufficient to maintain himself/herself. The participant was considered partially dependent if he/she had some personal income or any monetary benefit from the social welfare scheme, but which was not perceived to be sufficient to maintain himself/herself. The participant was classified as economically dependent if there was no personal income or monetary benefit from any social welfare scheme and s/he was totally dependent on other family members [14]. Living arrangement was categorised as living alone, living with spouse only, living with spouse and children or with son’s family, living with daughter’s family or distant relative or others [28]. Marital status was categorised as currently married and never married or divorced or widowed or separated [28,29]. We measured disability using WHODAS 2.0. This instrument was translated and validated in Hindi, the local language. WHODAS 2.0 is a cross-culturally applicable, reliable and valid tool for measuring disability [22]. It consists of six domains namely cognition, mobility, self-care, getting along, life activities, and participation. This scale was developed to reflect the concept of International Classification of Functioning, Disability and Health (ICF). The Cronbach’s alpha for this scale is 0.94 [22].

Data were first collected onto paper. All the interviews were checked by the Principal Investigator for completeness and coherence before data entry. Data were entered in Epi Info 7. The methods enumerated in the manual for WHODAS 2.0 were used for calculating the summary scores. The summary score ranges from 0 to 100, where 0 being no disability, and 100 being fully disabled. Elderly persons with summary score greater than 40 were categorised as disabled [20]. Prevalence of disability was reported as a proportion with 95% confidence interval (95% CI). We described the participant’s demographic and socioeconomic characteristics with a proportion or mean with standard deviation wherever applicable. WHODAS 2.0 summary scores were divided into equal quartiles to find the association between various factors. Multinomial logistic regression was carried out between the quartiles of WHODAS 2.0 summary scores and associated factors. The strength of association was reported as Relative Risk Ratio (RRR). Factors with significant association (p <0.05) in the crude model were included in the multivariable multinomial logistic regression. These analyses were carried out in the statistical software package STATA version 11.

The Ethics Committee of the All India Institute of Medical Sciences, New Delhi, granted ethical approval for conduct of the study. All participants were informed about the purpose of the study, and were provided with an information sheet in Hindi. Written informed consent was obtained from all participants. Participants found with any health problem were provided appropriate guidance or referral.

This study was funded by the Intramural Research Grant of All India Institute of Medical Sciences, New Delhi, India.

## Results

Of the randomly selected 1006 elderly persons, 931 interviews were completed. There were 17 refusals, and 58 elderly persons could not be contacted even after three visits to their homes. Response rate was 92.5%. There were 515 (55.3%) females and 416 (44.7%) males (Table 1). Mean (SD) age of the participants was 67.5 (6.8) years. There were 348 (37.4%) participants in the age-group of 60–64 years. Forty nine participants (5.3%) had completed secondary school, while 557 (59.8%) participants were illiterate. There were 571 (61.3%) currently married participants, and 837 (89.9%) participants lived in an extended family. At present, 770 (82.7%) participants were homemakers. In their past occupation, 309 (33.2%) participants were in government or private services. There were 448 (48.1%) partially economically dependent participants, and 748 (80.3%) participants belonged to Below Poverty Line (BPL) category. Of the total participants, 773 (83.0%) lived with their spouse and children or with son’s family. Eight hundred and eighty- two (94.7%) participants lived in their own house.

**Table 1:** Distribution of participants by socio-demographic characteristics (N = 931).

Characteristics	Number (n)	Percentage (%)
**Age group (years)**		
60–64	348	37.4
65–69	242	26.0
70–74	189	20.3
75 and above	152	16.3
**Sex**		
Male	416	44.7
Female	515	55.3
**Educational level**		
Illiterate	557	59.8
Primary	152	16.3
Middle	88	9.5
High	85	9.1
Secondary and above	49	5.3
**Type of family**		
Single member and Nuclear Family	94	10.1
Extended Family	837	89.9
**Marital status**		
Never married/divorced/widowed/separated	360	38.7
Currently married	571	61.3
**Past Occupation**		
Home maker	276	29.7
Govt. and Private Services	309	33.2
Business	145	15.6
Labourer and others	201	21.6
**Economical dependency status**		
Dependent	232	24.9
Partially dependent	448	48.1
Independent	251	27.0
**Living children**		
No children	12	1.3
Either son(s) or daughters(s) only	182	19.6
Both son and daughter	737	79.2
**Living arrangement**		
Living alone	31	3.3
Living with spouse only	74	8.0
Living with spouse and children or with son’s family	773	83.0
Living with daughter’s family or distant relative or others	53	5.7
**Ownership of house**		
Own house	882	94.7
Rented house	49	5.3

**Fig 1:**
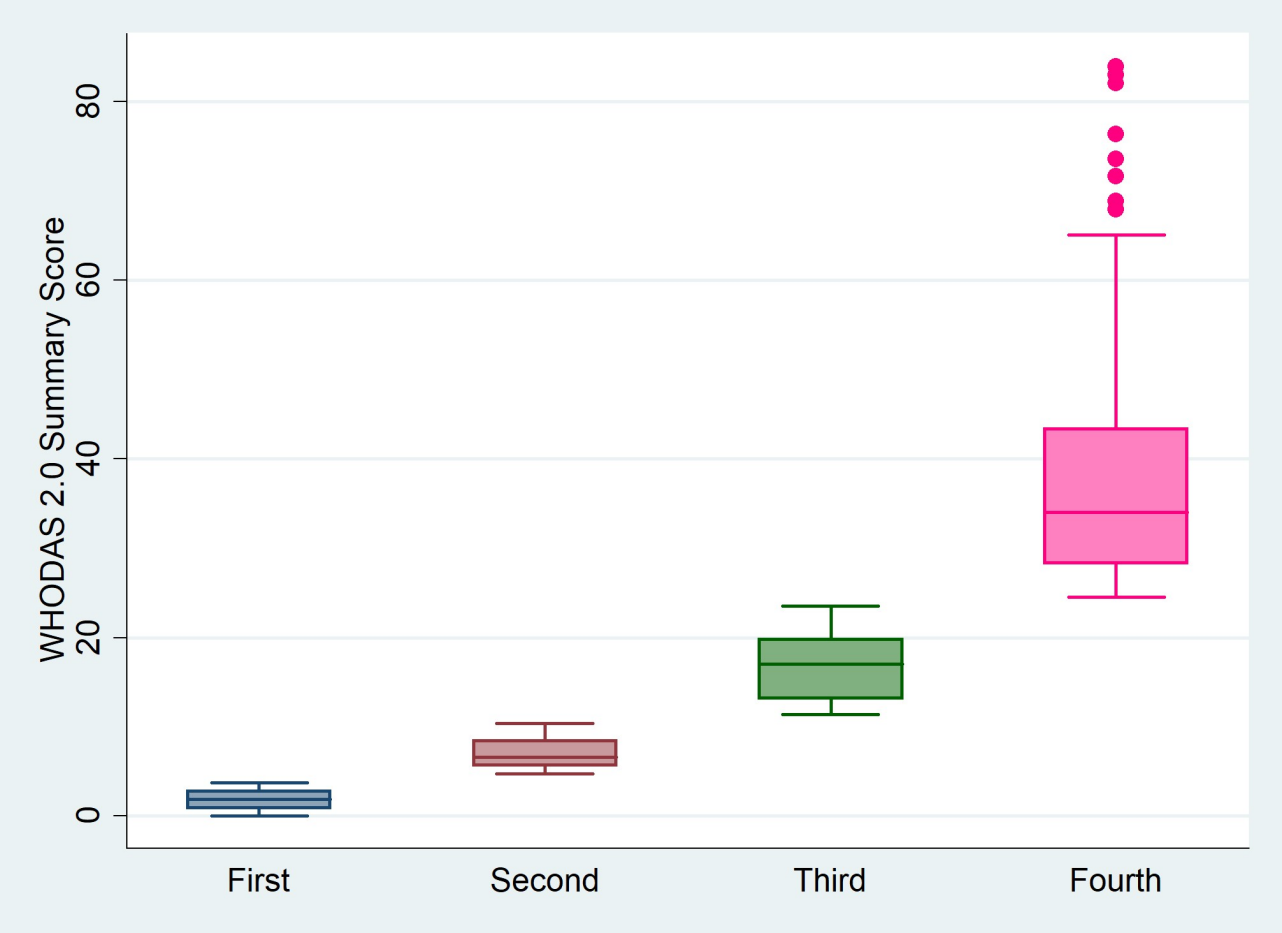
Distribution of the WHODAS 2.0 summary scores among the quartiles. Median of the First, Second, Third and Fourth quartile were 1.9, 6.6, 17.0 and 34.0 respectively.

The WHODAS 2.0 summary score was distributed with an interquartile range of 3.8 to 23.6 and median of 10.4. The prevalence of disability was 7.4% (95% CI 5.8% - 9.3%). The cut off at summary score of > 40, was used based on the Global Report on Disability, 2011 [20]. A community-based study by Almazen et al. categorised WHODAS 2.0 summary scores as no disability (0–4), mild disability (5–24), moderate disability (25–49), severe disability (50–95) and extreme disability (96–100) [30]. The corresponding prevalence figures for no disability, mild disability, moderate disability, severe disability in our study were 28%, 49%, 19.2%, and 3.8% respectively. No participant was extremely disabled. WHODAS 2.0 summary scores categorised into equal quartiles had median of 1.9, 6.6, 17.0 and 34.0 in the first, second, third and fourth quartiles respectively (Fig 1).

Increase in the proportion of female participants were observed in the ascending order of the quartiles and; it was found to be statistically significant (Table 2). There was a statistically significant difference in the number of participants among the quartiles of WHODAS 2.0 summary scores in the age group of the participants, level of education, type of family, marital status, present and past occupation, living arrangement and economic status of the family.

**Table 2:** Distribution of participants among the quartiles of WHODAS 2.0 disability score by socio-demographic characteristics.

Characteristics	First quartile (Q1) n (%)	Second quartile (Q2) n (%)	Third quartile (Q3) n (%)	Fourth quartile (Q4) n (%)	*p* value^a^
**Age group (years)**					
60–64	113 (32.5)	89 (25.6)	84 (24.1)	62 (17.8)	<0.001
65–69	75 (31.0)	60 (24.8)	59 (24.4)	48 (19.8)	
70–74	47 (24.9)	44 (23.3)	54 (28.6)	44 (23.3)	
75 and above	26 (17.1)	17 (11.2)	35 (23.0)	74 (48.7)	
**Sex**				
Male	170 (40.9)	94 (22.6)	70 (16.8)	82 (19.7)	<0.001
Female	91 (17.7)	116 (22.5)	162 (31.5)	146 (28.4)	
**Educational level**				
Illiterate	114 (20.5)	123 (22.1)	160 (28.7)	160 (28.7)	<0.001
Primary	50 (32.9)	32 (21.1)	33 (21.7)	37 (24.3)	
Middle	33 (37.5)	23 (26.1)	17 (19.3)	15 (17.1)	
High	35 (41.2)	22 (25.9)	20 (23.5)	8 (9.4)	
Secondary and above	29 (59.2)	10 (20.4)	2 (4.1)	8 (16.3)	
**Type of family**				
Single member and Nuclear Family	28 (29.8)	32 (34.0)	18 (19.2)	16 (17.0)	0.018
Extended Family	233 (27.8)	178 (21.3)	214 (25.6)	212 (25.3)	
**Marital status**					
Never married/divorced/ widowed/separated	184 (32.2)	137 (24.0)	128 (22.4)	122 (21.4)	<0.001
Currently married	77 (21.4)	73 (20.3)	104 (28.9)	106 (29.4)	
**Past Occupation**					
Home maker	52 (18.8)	64 (23.2)	82 (29.7)	78 (28.3)	<0.001
Govt. and Private Services	120 (38.8)	68 (22.0)	59 (19.1)	62 (20.1)	
Business	49 (33.8)	31 (21.4)	30 (20.7)	35 (24.1)	
Labourer and others	40 (19.9)	47 (23.4)	61 (30.4)	53 (26.4)	
**Economical dependency status**					
Dependent	64 (27.6)	52 (22.4)	58 (25.0)	58 (25.0)	0.767
Partially dependent	119 (26.6)	103 (23.0)	120 (26.8)	106 (23.7)	
Independent	78 (31.1)	55 (21.9)	54 (21.5)	64 (25.5)	
**Living children**					
No children	3 (25.0)	4 (33.3)	2 (16.7)	3 (25)	0.962
Either son(s) or daughter(s) only	49 (26.9)	40 (22.0)	45 (24.7)	48 (26.4)	
Both son and daughter	209 (28.4)	166 (22.5)	185 (25.1)	177 (24.0)	
**Living status**					
Living alone	7 (22.3)	12 (38.7)	7 (22.6)	5 (16.1)	0.008
Living with spouse only	19 (25.73)	24 (32.4)	17 (23.0)	14 (18.9)	
Living with spouse and children or with son’s family	225 (29.1)	168 (21.7)	194 (25.1)	186 (24.1)	
Living with daughter’s family or distant relative or others	10 (18.9)	6 (11.3)	14 (26.4)	23 (43.4)	
**Ownership of house**					
Own house	246 (27.9)	199 (22.6)	220 (24.9)	217 (24.6)	0.975
Rented house	15 (30.6)	11 (22.5)	12 (24.5)	11 (22.5)	

^a^ *p* values were calculated using the Chi Square test

In the multinomial logistic regression model, the first quartile of WHODAS 2.0 summary scores was considered as the reference or base outcome. In the crude model, compared to males, females had increased risk of having higher disability scores in the second (Relative Risk Ratio (RRR) = 2.3, 95% CI 1.6–3.3), third (RRR = 4.3, 95% CI 3.0–6.3) and fourth (RRR = 3.2, 95% CI 2.3–4.8) quartile of WHODAS 2.0 summary scores (Table 3). Participants aged 70–74 years had increased risk of higher disability scores in the fourth (RRR = 1.7, 95% CI 1.0–2.8) quartile compared to 60–64 years old. Almost two and five times increased risk of having higher disability scores in the third (RRR = 1.8, 95% CI 1.0–3.2) and fourth (RRR = 5.2, 95% CI 3.0–8.9) quartile was found among the elderly aged 75 years and above compared to 60–64 years. Illiterate participants had higher disability scores in all three quartile and it is statistically significant in the second (RRR = 20.4, 95% CI 4.8–87.0) and third (RRR = 5.1, 95% CI 2.2–11.5) quartile. Currently married participants had higher disability scores in the second (RRR = 1.9, 95% CI 1.3–2.8) and third (RRR = 2.1, 95% CI1.4–3.0) quartile compared to the never married/divorced/widowed/separated. Participants who were in business or in government and private service had almost 50% decreased risk of having higher disability scores in the second, third and fourth quartile.

**Table 3:** Crude multinomial logistic regression models of factors associated with quartiles of WHODAS 2.0 summary scores.

Covariates	Second quartile (Q2)			Third quartile (Q3)			Fourth quartile (Q4)		
	RRR^a^	95% CI	P Value	RRR	95% CI	P Value	RRR	95% CI	P Value
**Sex**									
Male	Reference			Reference			Reference		
Female	2.3	1.6–3.3	<0.001	4.3	3.0–6.3	<0.001	3.3	2.3–4.8	<0.001
**Age (years)**									
60–64	Reference			Reference			Reference		
65–69	1.0	0.7–1.6	0.944	1.0	0.7–1.6	0.802	1.2	0.7–1.9	0.527
70–74	1.2	0.7–2.0	0.495	1.5	1.0–2.5	0.077	1.7	1.0–2.8	0.042
75 & above	0.8	0.4–1.6	0.587	1.8	1.0–3.2	0.045	5.2	3.0–8.9	<0.001
**Educational level**									
Secondary & above	Reference			Reference			Reference		
High	3.1	1.5–6.7	0.003	8.3	1.8–38.4	0.007	0.8	0.3–2.5	0.737
Middle	1.8	0.8–4.3	0.151	7.5	1.6–35.1	0.011	1.6	0.6–4.4	0.324
Primary	2.0	0.8–4.9	0.123	9.6	2.1–42.8	0.003	2.6	1.1–6.5	0.030
Illiterate	1.8	0.7–4.5	0.189	20.4	4.8–87.0	<0.001	5.1	2.2–11.5	<0.001
**Type of family**									
Single member and Nuclear Family	Reference			Reference			Reference		
Extended Family	0.7	0.4–1.2	0.146	1.4	0.8–2.7	0.260	1.6	0.8–3.0	0.155
**Marital status**									
Never married/divorced/widowed/separated	Reference			Reference			Reference		
Currently married	1.3	0.9–1.9	0.224	1.9	1.3–2.8	<0.001	2.1	1.4–3.0	<0.001
**Past Occupation**									
Labourers and others	Reference			Reference			Reference		
Business	0.5	0.3–1.0	0.049	0.4	0.2–0.7	0.003	0.5	0.3–1.0	0.043
Govt. and Private service	0.5	0.3–0.8	0.006	0.3	0.2–0.5	<0.001	0.4	0.2–0.7	<0.001
Home maker	1.0	0.6–1.8	0.871	1.0	0.6–1.8	0.901	1.1	0.7–1.9	0.653
**Economical dependency status**									
Dependent	Reference			Reference			Reference		
Partially dependent	1.2	0.8–1.9	0.355	1.5	0.9–2.2	0.086	1.1	0.7–1.7	0.703
Independent	1.2	0.7–1.9	0.581	1.3	0.8–2.2	0.288	1.1	0.7–1.8	0.688
**Living children**									
No children	Reference			Reference			Reference		
Either son or daughter	0.6	0.1–3.0	0.536	1.4	0.2–8.6	0.732	1.0	0.2–5.1	0.980
Both son and daughter	0.6	0.1–2.7	0.502	1.3	0.2–8.0	0.758	0.8	0.2–4.2	0.840
**Living status**									
Living alone	Reference			Reference			Reference		
Living with spouse only	0.7	0.2–2.2	0.590	0.9	0.3–3.1	0.860	1.0	0.3–3.9	0.964
Living with spouse and children or with son’s family	0.4	0.2–1.1	0.088	0.9	0.3–2.5	0.785	1.2	0.4–3.7	0.806
Living with daughter’s family or distant relative or others	0.4	0.1–1.4	0.135	1.4	0.4–5.3	0.619	3.2	0.8–12.6	0.094
**Ownership of house**									
Own house	Reference			Reference			Reference		
Rented	0.9	0.4–2.0	0.810	0.9	0.4–2.0	0.780	0.8	0.4–1.8	0.651

^a^RRR: Relative Risk Ratio

**Fig 2:**
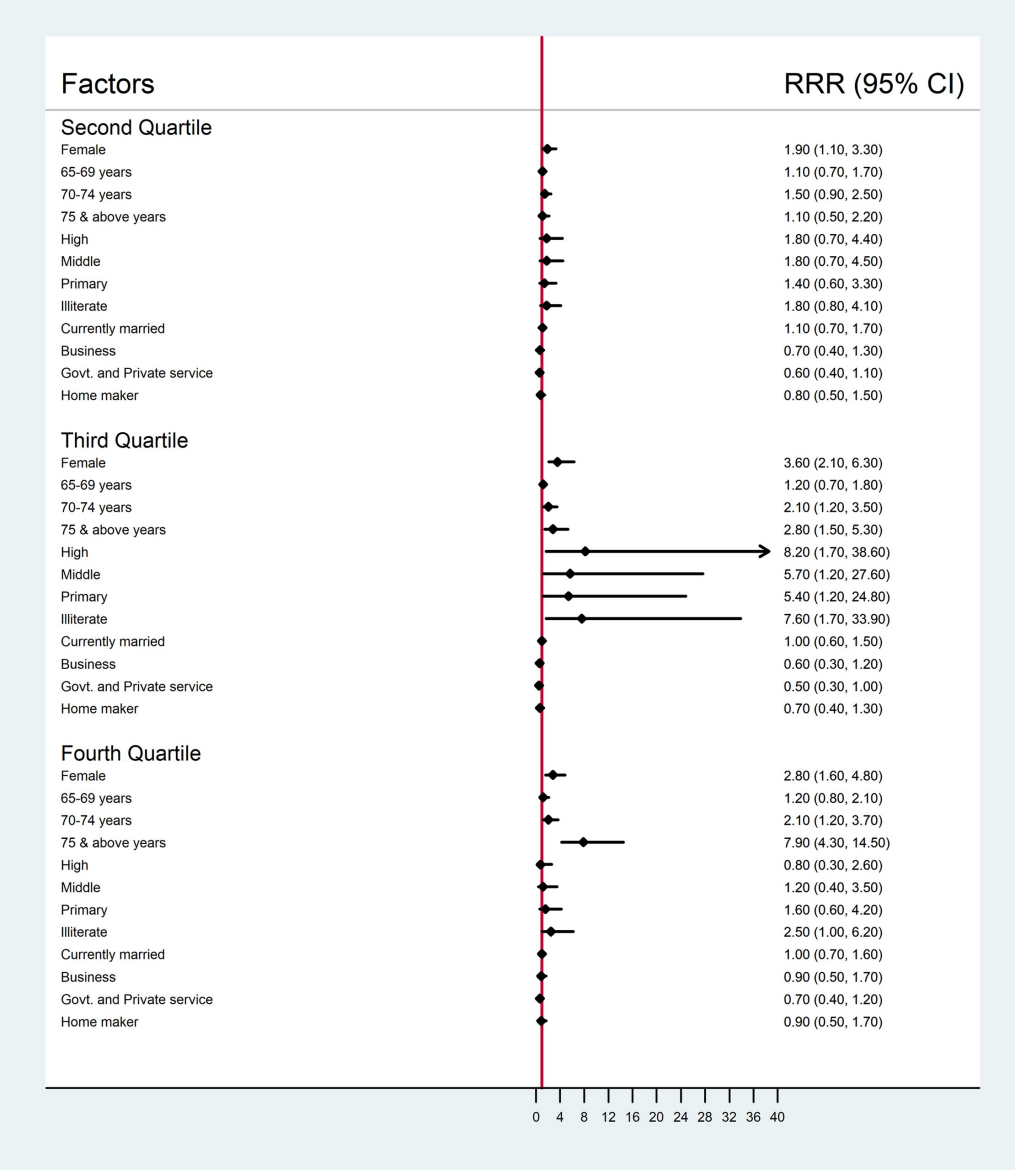
Factors associated with quartiles of WHODAS 2.0 summary scores by multinomial logistic regression analysis.

In the multivariable model, female sex, elderly aged 70 years and above and illiterate to those studied up to high school were associated with increased risk of higher WHODAS 2.0 disability scores (Fig 2). Participants who were in government or private service had 50% decreased risk of having higher disability scores in the second quartile, whereas in the third quartile females, elderly 70 years and above and only illiterate were having significantly increased risk of disability.

## Discussion

The prevalence of disability among elderly persons in our study was 7.4% (95% CI 5.8% - 9.3%), which was higher than the reported prevalence of disability by Census 2011 and NSSO 2002 [5,8]. This could be due to the inclusion of social and contextual factors which influence the level of disability. Among the studies that used WHODAS 2.0, the prevalence of disability varied based on the summary scores cut-offs used. A community-based cross-sectional study using WHODAS 2.0 was conducted among elderly persons ≥ 60 years in Pune by Sinalkar et al [31]. In this study, the WHODAS 2.0 summary score >4 was considered as disabled, and the reported prevalence of disability was 70.4%. In the study by Virues et al among elderly persons aged ≥ 75 years, disability was categorised as No disability (0–4), Mild disability (5–24), Moderate disability (25–49), Severe/Extreme disability (50–100); the corresponding age-adjusted disability prevalence figures were 39.17%, 15.31%, and 10.14% for mild, moderate, and severe/extreme disability, respectively [32]. Similar categories were used by Blazquez et al. in their study among persons aged ≥ 50 years and above in Spain [33]. It reported 51.5%, 28.9%, 16.1% had mild, moderate and extreme/severe disability, respectively. In our study, the corresponding figures for mild, moderate, severe, and extreme disability being 28.0%, 49%, 19.2% and 3.8%. Since disability is complex and has evolving concepts, different definitions and measurement scales were used in ascertaining disability in various studies. Usage of these different measurement scales may explain the variability in the prevalence of disability. By using WHODAS 2.0 for measuring disability, comparisons can be made between different populations. In all the above studies, females experienced higher disability than males, which is similar to our study findings. Almazan et al., Sinalkar et al, Virues et al., and Blazquez et al. reported higher disability among persons aged ≥ 70 years [30–33] Sinalkar et al. and Almazan et al reported that illiterate participants experienced higher disability than those who were literate [30,31]. Our study also reported an increase in disability scores as age increased. Disability levels decreased when the participants were literate and their past occupation was in government or private service. Gerontological studies found that elderly persons living with adult children, who are economically stable, had decreased stress and disability. Our study could not establish such an association. This could due to the low socio-economic status of the population in our study area [29]. A study by Gupta et al did not find any significant association between socioeconomic status and disability [13]. Economic dependency was not significantly associated with disability in the study by Gupta et al [14]. A study by Joshi et al found higher disability in the rural areas than in urban areas [18].

Strengths of the study were its community-based study design and good response rate. Data collected by specially trained interviewers increased the reliability of information. We have excluded individuals who cannot communicate and comprehend, this could have underestimated the disability prevalence. Being a cross-sectional study temporality of the findings could not be established and the findings are generalizable only to elderly persons of urban areas.

## Conclusion

The prevalence of disability among elderly persons in this resettlement colony was 7.4%. Disability increased with increasing age and was higher in female sex among elderly persons in urban areas. Elderly persons are more prone to non-communicable and communicable diseases resulting in disability. Policies for the welfare of elderly persons should identify and manage the conditions that lead to disability. Under the National Programme for Health Care of the Elderly, it is envisaged that rehabilitation units shall be established at the Community Health Centres. However, it is essential that comprehensive health care services should be provided to address geriatric disability at the community level. Providing learning and working opportunities for the young population, especially for females could reduce disability during old age. Longitudinal studies measuring disability using the bio-psycho-social model of the ICF would help the government and other non-governmental agencies to cater to the growing needs of the disabled elderly persons.
