# Correction: Disability and its association with sociodemographic factors among elderly persons residing in an urban resettlement colony, New Delhi, India

**DOI:** 10.1371/journal.pone.0224494

**Published:** 2019-10-23

**Authors:** Anil Kumar Goswami, Sathiyamoorthy Ramadass, Mani Kalaivani, Baridalyne Nongkynrih, Shashi Kant, Sanjeev Kumar Gupta

The second author’s name is spelled incorrectly. The correct name is: Sathiyamoorthy Ramadass. The correct citation is: Goswami AK, Ramadass S, Kalaivani M, Nongkynrih B, Kant S, Gupta SK (2019) Disability and its association with sociodemographic factors among elderly persons residing in an urban resettlement colony, New Delhi, India. PLoS ONE 14(9): e0222992. https://doi.org/10.1371/journal.pone.0222992

## References

[1] Goswami AK, S. R, Kalaivani M, Nongkynrih B, Kant S, Gupta SK (2019) Disability and its association with sociodemographic factors among elderly persons residing in an urban resettlement colony, New Delhi, India. PLoS ONE 14(9): e0222992 10.1371/journal.pone.0222992 31550291 PMC6759158

